# Editorials

**Published:** 1908-12

**Authors:** 


### EDITORIALS

The Business Office of the JOURNAL-RECORD is 1 1-2 to 5 1-2 South Broad Street.
The Editorial Office is 1014-15 Century Building.
Address all Business Communications to *Journal-Record of Medicine*, 1 1-2 to 5 1-2 South Broad Street.
Make remittances payable to *THE JOURNAL-RECORD OF MEDICINE.*
On matters pertaining to the Editorial and Original Communications, address Edgar G. Ballenger, M. D., Atlanta, Ga.
Reprints of Original Articles will be furnished at cost price. Requests for the same should always be made in THE MANUSCRIPT.
We will present, postpaid, on request, to each contributor of an original article, twenty (20) marked copies of THE JOURNAL-RECORD OF MEDICINE containing such article.

### A SCHEDULE OF MEDICAL FEES.

Early in the year which is approaching its end the Fulton County Medical Society appointed two committees, called respectively, the Collection Committee and the Regulation of Fees Committee. The work of the former obtained an unexpected notoriety from being scented by the keen nose of a newspaper young gentleman whose official armament includes an exuberant and uncurbed fancy unclouded by any special development of the organs of either gratitude or veneration and untramelled by the least caring as to the probable effect of his exploration of what promised an hour's sensation. In a brilliant effort to be funny and entertaining, the profession was represented as suddenly seized by a craze for money-getting which had cut it off from that charity with which till now it had overflowed into the laps of many even of those who were totally undeserving. But the publicity thus produced, in spite of and perhaps to some extent on account of the manner of its production had on the whole a beneficial effect and evoked from the laity many expressions of sympathy with the position of the profession in regard to its innovation The work of the committee on the Regulation of Fees is not complete, and will doubtless be carried on into next year. It is interesting in the mean time to observe that Dr. J. N. McCormack, the official organiser of the American Medical Association, has recently produced "a general plan for a Schedule of Medical Fees" whose methods very much resemble those of the Fulton County Society. He says, "I am advising that the profession in each county or city consider the advisability of arranging for systematic monthly collections, with a carefully selected business representative, and a centrally located medical collector's office, the collector to be under bond, and on a definite salary, and with authority to appoint as many assistants as may be necessary, for whom he is responsible, very much as sheriffs and city collectors do." Dr. McCormack lays stress upon the present general inadequacy of medical fees and suggests a schedule which could be modified to suit local conditions, and which should be framed according to the cost of living and of modern medical equipment, which have respectively doubled and quadrupled in recent years. These fees, he thinks, and the system in vogue, should be made as public as possible.

Dr. McCormack has had exceptional opportunity of studying "rate-cutting and cheap doctors," and has taken full advantage of it. His conclusions are that "they charged less for their services because they honestly knew better than any one else did or could, that they were worth less than their competitors, and that this was their only chance to obtain and hold practice," and, he continues, "the fault is far more with the schools which pretend to educate these men than with them." It is undoubtedly true that the medical profession, by permitting the entrance through some of its portals to be disgracefully easy, attracts to its ranks many men mentally and morally incapable of appreciating the sanctity of their "calling."

S.

### A MODEL REPORT.

Perhaps many have inquired in their own minds how writers of monumental works on various subjects—particularly medical subjects—managed to gather such a wealth of material and prepare it for assimulation by the average reader. Perhaps a few know, that as a rule, a large part, if not the largest part, of such material is culled from carefully compiled and accurate reports. And in proportion as the reports are faithful and scientific reflections of actual conditions, in just such proportion will our lately revised and popularly accepted standard text-books be correct.

The Fourth Annual Report of the Phipps Institute for the study, treatment and prevention of Tuberculosis is a most admirable example of an excellent report. A careful study of this report will repay not only the medical author in search of accurate material, but will richly reward any student of medicine who is desirous of first hand information in regard to the protean phases of this many sided disease. If one is able to draw deductions from careful analyses, a wealth of practical information is contained in the 404 pages of the report.

Certain portions of the report are of special practical interest. Besides being an excellent model for future similar reports from like institutions, the clinical and sociological reports of the year by the able director, Dr. Lawrence F. Flick, contains a wealth of useful and suggestive information. After each statistical table Dr. Flick comments on his figures in a very clear and convincing manner, frequently dealing in an interesting way with debated questions. His comments on hoarsness in tuberculosis on page 71 will be appreciated by throat specialists. Another point of interest is the presence of pain in tuberculosis. Consumption is often thought of as a painless disease, and yet these statistics show that of the seven hundred and thirty-four new cases for the current year, over eighty per cent. suffered pain thus rendering it, after cough, the most frequent symptom of the disease. Still another matter of great interest which is as yet little understood, is that of disturbances of the temperature, pulse and respiration. The tables giving the results of treatment in patients with pulse above and below 100 and with respiration above and below 30, when first seen, are very instructive. A pulse rate of 100 or over and a respiratory rate of 30 or over are distinctly unfavorable prognostic signs, the latter being relatively more serious than the former according to the statistics.

The report on the blood findings in tuberculosis based on a study of 83 cases is of interest. Dr. Craig, who makes this report concludes the leucocytosis in tuberculosis can give no very definite information either in regard to the usual complications or the presence of cavity formation. The most constant finding was a chloro-anaemia present in practically all cases.

A portion of the report which is of great interest is a study by Dr. Geo. Bacon Wood on the Importance of the Upper Respiratory tract in the Etiology of Cryptogenic Infections especially in relation to Pleuritis. One result which his study shows is that of thirty-four cases of tuberculosis of the lungs studies, "the tonsils of twenty-nine showed absolutely typical lesions of tuberculosis." This study is well worth careful perusal.

Doubtless no more helpful article to the student of physical diagnosis could be found anywhere than that portion of the report dealing with the comparison of the pathological findings with the recorded clinical signs in nine cases of tuberculosis of the lungs which came to complete autopsy. This report is made by Dr. Joseph Walsh. A careful perusal of the detailed reports of each case will give one a better idea of the practical value of the ordinary signs of pulmonary conditions than can be found in any text-book.

Here one can study out for himself by this admirable check system the successes as well as the failures of the physical signs.

The entire report is of great practical value and scientific interest and should shed much light on the intricate problems of the disease with which it deals.

L. M. G.

### PELLAGRA IN THE SOUTHERN STATES.

The recent outbreak of Pellagra throughout the Southern States has aroused much interest and every physician should be familiar with the nature and treatment of this disease. Searcy* in a recent article discusses in a most interesting manner the history and characteristics of this dangerous malady. He defines Pellagra (*pelle,* skin, *agra,* rough) as a condition induced by continuously eating damaged maize or Indian corn, manifested by disorders of the nervous system, derangement of the digestive system, and an erythema of the skin in certain parts of the body. Although the name is taken from the skin symptom this is regarded as the least important. Pellagra has been known in Spain since 1755, following the introduction of maize from America in about 1700.

Harris and Searcy reported the first cases that were known to have originated in the United States. The disease is quite similar to ergotism and Searcy thinks corn smut is the cause of pellagra. He gives the symptomatology as follows:

*The acute form* first shows itself in a general run-down condition, lassitude, loss of flesh, weakness, and dyspepsia. This condition may be a few weeks or months in developing. It cannot be definitely estimated, as it depends on the amount of the toxin ingested and the condition of the patient previously.

The characteristic skin lesions and those arising from the alimentary tract appear about the same time. The patient begins to have a profuse flow of saliva, the whole mouth looks red and sore, the tongue often looks denuded of epithelium, and there are digestive disturbances, tenderness over the stomach, and sooner or later, diarrhea.

About the same time there appears a deep red congestion or erythema on one or more of the exposed dorsal surfaces of the body, as the backs of the hands and lower forearms, dorsal surface of the feet, back of the neck, the cheeks, and occasionally the genitals.

The skin in these regions takes on a dark red of congested hue, with no pain and but slight burning or itching sensations; most usually it is a dead feeling, with all sensations dulled in these parts. In a few days vesicles or bullae may form, and later break and peel off, leaving a raw, weeping surface, which, if the patient survives, gradually heals, leaving a thin, silk-like skin. When vesicles and bullae do not form, the skin dries and desquamates, leaving a rough, mealy skin, which slowly smooths over.

The skin lesions are very symmetrical. When on the back of one hand or foot or cheek you will find a like patch on the other. It rarely extends more than six inches up the forearm. It sometimes is found on the elbows, and in about 10 per cent. of the cases on the genitals. I have never seen on such flexor surfaces as the palm, the bend of the elbow, axilla, or soles.

Along with the salivation, diarrhea, skin lesions, etc., come the mental and spinal symptoms, but these are not so marked in the early acute attacks, as they develop when the disease becomes chronic. Even at first, however, there is some dullness and depression noticed, very much as you find in any severe intoxication. This dullness and depression seem more marked among the insane cases than those outside the hospital.

The other nervous symptoms are pain or tenderness on both sides of the spinal column, especially in the dorsal region, and at first irregular, exaggerated patellar reflexes, but later dulled or absent reflexes. There is usually also insomnia of marked degree, and there is tenderness over the stomach, and in women pain or tenderness over the uterus.

The arterial tension is at first increased and the pulse rate a little above normal, and this goes up or down with the temperature. The temperature in uncomplicated cases rarely goes above a degree and a half above normal, and often gets subnormal. The urine often shows a higher specific gravity than normal, due probably to the fact that the patient takes very little fluid, and so there is a diminished amount of urine passed. The blood shows anaemia, but nothing characteristic.

Bacteriological examinations have proven negative, and examinations of the stools of the Mt. Vernon patients have not shown a single case of hook-worm infection.

The fatal cases may prove rapidly so in a few days from the time they take to bed, or they may run on several weeks with the salivation, diarrhea, sore hands or feet, etc., the pulse gradually getting weaker and the patient finally dying from general weakness.

When recovery follows, convalescence is very slow; the patient remains weak and more or less dull a month or more, and for several months remains below normal.

The mild cases may never go to bed, but sit about in a dull, listless manner, look weak and emaciated, with red tongue and mouth, spitting a great deal, the hands or feet or some of these dorsal surfaces showing the dry, mealy skin or the red, weeping surface, giving little or no pain or discomfort, and, lastly, with more or less diarrhea.

*The Chronic Forms.*—These cases will usually give a history of attacks during previous summers. With every attack the erythema leaves a little more pigment and thickening behind it until chronic atrophy takes place, showing where the eruption has been. The affected skin will then look wrinkled, like that of an old man. In the later attacks fewer vesicles form and the eruption is mostly of the dry, scaly type.

During the first, and even the second, year of pellagara in an individual of average intelligence no definite mental symptoms are noticed, but after that he becomes decidedly stupid and somewhat melancholic, taking little interest in anything beyond his food and sleep. As the disease progresses these mental symptoms may become more definite and there may also be hallucination and delusions of such prominence as to cause the patient to be confined in an asylum.

The usual ending of these cases showing such marked mental symptoms, and which do not respond to treatment, is in secondary dementia (Sandwith).

These chronic cases may, after they have become bedridden, develop contractures of the fingers or the lower limbs, or even paraplegia (Sandwith). They always retain control of the bladder and rectum and rarely have bedsores.

During the exacerbations of the chronic cases the red mouth and tongue may not be so pronounced as at first, but there is the weakness, emaciation, disturbance of digestion and always more or less diarrhea.

The prognosis is always grave, death occuring in more than half the cases; recovery is very slow if the patient survives.

The treatment consists chiefly in removing from the patients food all corn products and substituting a good liquid and good hygienic surroundings, but not bright sunlight. Searcy recommends arsenic in the form of atoxyl in 1 1-2 grain doses hypodermically once a week and gradually increased to 2 grains at a dose.

### A NATIONAL DEPARTMENT OF PUBLIC HEALTH.

Dr. Charles A. I. Reed, of Cincinnati, in an address* delivered before the New York Academy of Medicine makes a strong appeal for a National Department of Public Health. He discusses the various causes of the failure of Congress to pass measures heretofore submitted and shows that many of them were premature in the sense that a public sentiment was not previously too radical—most legislation being built on previous legislation is therefore evolutional in character; other measures have failed because of a lack of medical men among our representatives. It is justly alleged that too little knowledge concerning public health has reached the people, most of it being discussed in medical societies and published in medical journals only. Reed advocates informing the public of our notable successes such as control of diseases in the Panama canal zone; the work of Finlay, Reed, Lazear and Carroll; how our health and life are being made secure by the efficient work of the Public Health and Marine-Hospital Service. He furthermore urges the importance of making more public information concerning the monetary loss from preventable diseases as tuberculosis, typhoid fever, etc.;

*Jour. A. M. A., November 28th, 1908.

of the importance of protecting our water sheds and of recognizing the constant menace from flies, musquitoes, etc.

The unorganized and scattered services can be made vastly more adequate if all the national government's effort be placed in one department under one head and similar laws be passed by all states so that we may have uniformity of sanitary laws, especially those that are co-extensive with all the states—this is to be obtained through a "council of states."

"Like laws for like conditions is the first requisite for the control for any national necessity by state legislation. And like laws by the states will probably not be enacted to any great extent until, under the initiative of the national government, the states shall send delegates to a representative body, a sort of council of states, which shall meet as a legislature or as Congress meets, in a session or sessions long enough to accomplish satisfactory results, and whose object shall be to formulate standard bills on various subjects and send them back to the legislatures for enactment. In this way, and in this way alone, can the states move with anything like satisfactory rapidity in meeting the crying demand for like laws for like conditions not only in regard to great sanitary problems such as I have been discussing, but in regard to many other problems that concern the economic and social welfare of all the people. This step should be taken without delay, for, even at the best, considerable time must elapse before results from this means can be realized.

But, aside from the special proposition that we are here discussing, some such step as I have outlined is imperatively demanded to conserve the states in their present integrity."

